# Genetic susceptibility of eight nonsynonymous polymorphisms in *HLA-DRB1* gene to hepatocellular carcinoma in Han Chinese

**DOI:** 10.18632/oncotarget.13111

**Published:** 2016-11-04

**Authors:** Yanhui Shi, Weiyu Zhai, Bin Wang, Dongmei Zhao, He Jin, Yuefei Wang, Jidong Zhang, Hongjun An, Zhongze Fu, Kun Zhao, Changzhu Lu

**Affiliations:** ^1^ Department of Gastroenterology, The First Hospital of Qiqihar City, Qiqihar, Heilongjiang, China; ^2^ Department of Pharmacy, The First Affiliated Hospital of Qiqihar Medical University, Qiqihar, Heilongjiang, China; ^3^ Department of Physiology, Qiqihar Medical University, Qiqihar, Heilongjiang, China; ^4^ Department of Cardiology, Hospital of Traditional Chinese Medicine of Qiqihar, Qiqihar, Heilongjiang, China

**Keywords:** hepatocellular carcinoma, human leukocyte antigen, non-synonymous polymorphism, genetic predisposition, Han Chinese

## Abstract

**Backgrounds and Objective:**

Mounting evidence suggests that human leukocyte antigen (HLA) plays a central role in anti-virus and tumor defense. To test whether genetic variation in *HLA*-*DRB1* gene, a key component of HLA system, can predict its predisposition to hepatocellular carcinoma (HCC), we thereby conducted an association study by genotyping 8 nonsynonymous polymorphisms in *HLA*-*DRB1* gene among 257 HCC patients and 264 controls.

**Results:**

All polymorphisms respected the Hardy-Weinberg equilibrium. The genotypes and alleles of rs17879599 differed significantly between patients and controls after Bonferroni correction (both *P* < 0.001), and the power to detect this significance was 94.4%. After adjusting for age, gender, smoking, drinking and hepatitis infection, the mutant allele of rs17879702 was significantly associated with an increased risk for HCC under additive (odds ratio [OR] = 2.12, 95% confidence interval [CI]: 1.20-4.02, *P* = 0.004) and dominant (OR = 2.51, 95% CI: 1.39–2.96, *P* = 0.004) models. Haplotype analysis indicated that haplotype A-T-C-T-G-C-T-A (alleles ordered by rs199514452, rs201540428, rs201614260, rs17879702, rs17880292, rs17879599, rs17424145 and rs35445101) was overrepresented in patients and enhanced predisposition to HCC (adjusted OR = 2.72, 95% CI: 1.24–5.78, *P* = 0.004). In cumulative analysis, carriers of 7–9 unfavorable alleles had a 2.41-fold (95% CI: 1.18–4.92, *P* = 0.016) increased risk for HCC after adjusting for confounding factors relative to those possessing 4 or less unfavorable alleles.

**Materials and Methods:**

Genotypes were determined by ligase detection reaction. HCC patients were newly diagnosed, histopathologically confirmed or previously untreated and controls were cancer-free.

**Conclusions:**

Our findings suggest an independent leading contribution of rs17879599 in the 2nd exon of HLA-DRB1 gene to HCC risk in Han Chinese.

## INTRODUCTION

As the most common form of human liver cancer, hepatocellular carcinoma (HCC) ranks as one of the world's prevalent malignancies, especially in China [1]. The rising incidence of HCC is mainly attributable to chronic hepatitis B and C virus infection [2]. Importantly, a familial clustering of HCC lends some credence to the involvement of a genetic component in the pathogenesis of HCC [3–6]. In view of the fact that loss of immune surveillance can stimulate the activation of various tumor cells [7, 8], it is clinically essential to interrogate whether lack of inherited putative protective alleles in human leukocyte antigen (HLA) class II genes can enhance predisposition to HCC risk. However, no general consensus in the literature has been reached on this claim because of considerable controversies over the relative predisposition of each allele to HCC [9].

Mounting evidence suggests that human HLA system plays a central role in anti-virus and tumor defense [10], and the onset and progression of HCC is closely correlated to this system, in particular a key component - HLA class II DRB1 gene (*HLA-DRB1*, ID: 3123) [11, 12]. It is widely recognized that down-regulation of host immune response represents a more critical signal in hepatocarcinogenesis [13]. Existing data from clinical epidemiology have suggested that *HLA*-DRB1 gene was significantly down-regulated in HCC patients with early intrahepatic recurrence relative to the relapse-free patients [14]. The genomic sequence coding *HLA-DRB1* gene is polymorphic, and there are thus far 147 validated alleles harboring this gene [15–17]. Systematic evidence from a meta-analysis of eight observational studies supported a susceptible impact of specific *HLA*-DRB1 gene alleles in the development of HCC [18]. To produce more information, we therefore designed a case-control association study in the Northeast of China to examine the predisposition of eight nonsynonymous bi-allelic polymorphisms in *HLA-DRB1* gene to HCC.

## RESULTS

Table 1 compares the baseline characteristics of 257 HCC patients and 264 controls. Controls were significantly older than patients (65.14 years vs. 58.75 years, *P <* 0.001) and had a lower proportion of male gender (64.77% vs. 79.38%, *P <* 0.001). In contrast, there was a higher proportion of smokers and hepatitis infectors in patients than in controls (both *P <* 0.001). No difference existed in the distributions of drinking and diabetes mellitus between the two groups. For HCC patients, the median values of alpha-fetoprotein (AFP), glucose intolerance (GI) and carcinoembryonic antigen (CEA) were 3.18 ng/mL, 9.76 U/mL and 2.21 ng/mL among 144, 122 and 129 patients, respectively.

**Table 1:** The baseline characteristics of study participants

Characteristics	Patients	Controls	*P*
Sample size	257	264	
Age (years)	58.75 (10.32)	65.14 (9.57)	< 0.001
Gender			< 0.001
Males	204	171	
Females	53	93	
Smoking	79.38%	64.77%	< 0.001
Drinking	36.19%	30.77%	0.274
Diabetes	8.53%	11.22%	0.450
Hepatitis infection	68.87%	7.14%	< 0.001
AFP (ng/mL) (*n*=144)	3.18 (1.79, 56.54)	n.a.	
GI (U/mL) (*n* = 122)	9.76 (4.47, 20.61)	n.a.	
CEA (ng/mL) (*n* = 129)	2.21 (1.26, 3.96)	n.a.	

Abbreviations: AFP, alpha-fetoprotein; GI, glucose intolerance; CEA, carcinoembryonic antigen; n.a., data not available. Continuous data are expressed as mean (standard deviation) or median (inter-quartile range: 25th percentile to 75th percentile). Categorical data are expressed as percentages. Two-group comparisons were conducted by the *t*-test for continuous data and by the χ^2^-test for categorical data.

Genotypes of all study polymorphism respected the Hardy-Weinberg equilibrium in controls at a significance level of 5%. The genetic distributions of eight study polymorphisms are presented in Table 2. After the Bonferroni correction for multiple testing (*P <* 0.05/8), significance was found for rs17879599 only in genotype and allele distributions between HCC patients and controls (both *P <* 0.001). The power to reject the null hypothesis of no allelic difference for rs17879599 between the two groups was estimated to be 94.4%. In addition, there was marginal significance for the allele distributions of rs17879702 (*P* = 0.007), with an estimated power of 77.3%.

**Table 2:** The comparison of the genotypes and alleles of eight study polymorphisms between HCC patients and controls

Polymorphisms	Class	WW	WM	MM	*P	M	*P*
rs199514452		AA	AT	TT		T	
c.31T > A	Patients	170	78	9	0.847	18.66%	0.837
	Controls	175	82	7		18.18%	
rs201540428		TT	TA	AA		A	
c.34A > T	Patients	163	84	10	0.754	20.23%	0.447
	Controls	159	93	12		22.16%	
rs201614260		CC	CG	GG		G	
c.84G > C	Patients	218	39	0	0.043	7.59%	0.039
	Controls	240	24	0		4.55%	
rs17879702		CC	CT	TT		T	
c.133C > T	Patients	201	52	4	0.021	11.67%	0.007
	Controls	230	32	2		6.82%	
rs17880292		GG	GA	AA		A	
c.256G > A	Patients	215	42	0	0.177	8.17%	0.673
	Controls	221	39	4		8.90%	
rs17879599		GG	GC	CC		C	
c.299G > C	Patients	129	113	15	< 0.001	27.82%	< 0.001
	Controls	177	76	11		18.56%	
rs17424145		TT	TC	CC		C	
c.341T > C	Patients	227	30	0	0.311	5.84%	0.277
	Controls	241	23	0		4.36%	
rs35445101		AA	AG	GG		G	
c.790T > C	Patients	213	43	1	0.247	8.75%	0.193
	Controls	210	49	5		11.17%	

Abbreviations: WW, homozygous wild genotype; WM, heterozygous genotype; MM, homozygous mutant genotype; M, mutant allele. *P* was calculated by the *Fisher's exact test for genotype comparisons and by the χ^2^-test for allele comparisons between patients and controls.

The risk prediction for HCC was explored under additive and dominant models of eight study polymorphisms with and without adjusting for age, gender, smoking, drinking and hepatitis infection (Table 3). The risk genotypes of rs17879702 and rs17879599 were associated with the significant risk of HCC, especially under dominant model after adjusting for age, gender, smoking, drinking and hepatitis infection (for rs17879702: odds ratio [OR] = 2.51, 95% confidence interval [CI]: 1.34–4.69, *P* = 0.004 and for rs17879599: OR = 1.89, 95% CI: 1.21–2.96, *P* = 0.005), and significance remained even after the Bonferroni correction.

**Table 3:** The prediction for HCC risk conferred by eight study polymorphisms with and without adjustment under both additive and dominant models

Polymorphisms	Additive model				Dominant model			
	OR (95% CI)	*P*	OR (95% CI)	P*	OR (95% CI)	*P*	OR (95% CI)	P*
rs199514452	1.03 (0.75–1.42)	0.835	1.08 (0.69–1.67)	0.624	1.01 (0.70–1.45)	0.973	1.06 (0.64–1.76)	0.708
rs201540428	0.89 (0.66–1.20)	0.444	0.90 (0.62–1.30)	0.578	0.87 (0.61–1.24)	0.453	0.91 (0.58–1.42)	0.672
rs201614260	1.79 (1.04–3.07)	0.035	1.99 (1.00–3.96)	0.051	1.79 (1.04–3.07)	0.035	1.99 (1.00–3.96)	0.051
rs17879702	1.78 (1.16–2.73)	0.009	2.12 (1.20–4.02)	0.004	1.88 (1.18–3.00)	0.008	2.51 (1.34–4.69)	0.004
rs17880292	0.91 (0.59–1.41)	0.674	0.86 (0.51–1.44)	0.563	1.00 (0.63–1.60)	0.987	0.96 (0.55–1.69)	0.899
rs17879599	1.72 (1.27–2.32)	< 0.001	1.63 (1.12–2.39)	0.012	2.00 (1.42–2.88)	< 0.001	1.89 (1.21–2.96)	0.005
rs17424145	1.38 (0.78–2.46)	0.265	1.32 (0.64–2.76)	0.453	1.38 (0.78–2.46)	0.265	1.32 (0.64–2.76)	0.453
rs35445101	0.77 (0.51–1.15)	0.198	0.96 (0.57–1.62)	0.893	0.80 (0.52–1.25)	0.331	1.02 (0.58–1.77)	0.958

Abbreviations: OR, odds ratio; 95% CI, 95% confidence interval

* *P* was calculated in a multivariate binary Logistic regression model after adjusting for age, gender, smoking, drinking and hepatitis infection.

**Figure 1:**
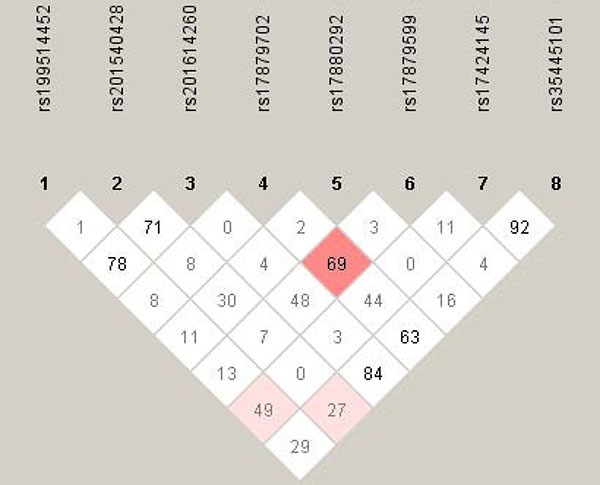
The linkage disequilibrium profiles of eight study polymorphisms The number in diamond represents the 100 × *D*' in the form of standard color scheme. The pink box denotes *D*' < 1 and LOD ≥ 2, and the while box denotes *D*' < 1 and LOD < 2.

Figure 1 displays the linkage disequilibrium profiles of eight study polymorphisms among all participants. The linkage magnitude between rs17879702 and rs17879599 was moderate as highlighted in the pink color (*D*' = 0.69, LOD = 20.34).

The estimated frequencies of common haplotypes (> 3% in all participants) between HCC patients and controls are summarized in Table 4. The most common haplotype was A-T-C-C-G-G-T-A (alleles in order of rs199514452, rs201540428, rs201614260, rs17879702, rs17880292, rs17879599, rs17424145 and rs35445101), which had a higher frequency in controls than in patients (40.28% vs. 35.89%, *P* = 0.013). After the Bonferroni correction (*P <* 0.05/7), only haplotype A-T-C-T-G-C-T-A, which was overrepresented in patients, differed significantly between the two groups (*P* = 0.003). When using the most common haplotype as the reference group, the risk prediction for HCC was significant for haplotype A-T-C-T-G-C-T-A after adjusting for age, gender, smoking, drinking and hepatitis infection (OR = 2.72, 95% CI: 1.24–5.78, *P* = 0.004).

**Table 4:** The frequencies of estimated haplotypes between patients and controls and their risk prediction for HCC risk with and without adjusting for potential confounders

Haplotype	Patients	Controls	*P*	OR (95% CI)	*P*	OR (95% CI)	P*
A-T-C-C-G-G-T-A	0.3589	0.4028	0.013	Reference group		Reference group	
T-T-C-C-G-G-T-A	0.0603	0.0926	0.265	0.85 (0.46–1.57)	0.609	1.00 (0.41–2.45)	0.983
A-A-C-C-G-G-T-A	0.0525	0.0861	0.066	0.83 (0.43–1.64)	0.599	0.85 (0.47–1.81)	0.617
A-T-C-C-G-C-T-A	0.0682	0.0467	0.192	2.19 (1.09–4.39)	0.028	1.88 (0.96–3.37)	0.174
A-T-C-T-G-C-T-A	0.0672	0.0502	0.003	1.78 (0.95–3.33)	0.072	2.72 (1.24–5.78)	0.004
A-T-C-C-A-G-T-A	0.0458	0.0501	0.658	0.85 (0.41–1.79)	0.672	0.83 (0.33–1.77)	0.606
A-A-C-C-G-G-T-G	0.0280	0.0525	0.201	0.56 (0.24–1.32)	0.187	0.73 (0.25–1.99)	0.545

Abbreviations: OR, odds ratio; 95% CI, 95% confidence interval. For each haplotype, alleles are arranged in order of rs199514452, rs201540428, rs201614260, rs17879702, rs17880292, rs17879599, rs17424145 and rs35445101.

* *P* was calculated in a multivariate binary Logistic regression model after adjusting for age, gender, smoking, drinking and hepatitis infection.

The cumulative impact of eight study polymorphisms on HCC risk is provided in Table 5. Genetic scores based on the number of unfavorable alleles were created and grouped in quartiles, with the genetic score of 4 or less unfavorable alleles as the reference group, which was frequently common in controls relative to in patients. Compared with this reference group, carriers of 7–9 unfavorable alleles had an approximate two-fold increased risk for HCC, especially after adjusting for age, gender, smoking, drinking and hepatitis infection (OR=2.41, 95% CI: 1.18–4.92, *P* = 0.016). The adjusted risk associated with per score and quartile increments in genetic score was significant, with the odds of HCC being 1.24 (95% CI: 1.07–1.44, *P* = 0.005) and 1.30 (95% CI: 1.06–1.60, *P* = 0.013), respectively.

**Table 5:** Genetic score analysis of eight study polymorphisms and the risk prediction for HCC with and without adjustment

Genetic score	Patients *n* (%)	Controls *n* (%)	OR (95% CI)	*P*	OR (95% CI)	P*
Quartiles:						
1–4	96 (37.36%)	130 (49.24%)	Reference group		Reference group	
5	68 (26.46%)	64 (24.24%)	1.44 (0.93–2.21)	0.098	1.40 (0.82–2.39)	0.211
6	51 (19.84%)	42 (15.91%)	1.64 (1.01–2.67)	0.045	1.52 (0.82–2.80)	0.184
7–9	42 (16.35%)	28 (10.60%)	2.03 (1.18–3.51)	0.011	2.41 (1.18–4.92)	0.016
Per score increment			1.23 (1.09–1.39)	0.001	1.24 (1.07–1.44)	0.005
Per quartile increment			1.27 (1.08–1.50)	0.004	1.30 (1.06–1.60)	0.013

Abbreviations: OR, odds ratio; 95% CI, 95% confidence interval

* *P* was calculated in a multivariate binary Logistic regression model after adjusting for age, gender, smoking, drinking and hepatitis infection.

## DISCUSSION

The HLA system is composed of a family of class I and class II genes within major histocompatibility complex on chromosome 6 [19]. DRB1 is a member of HLA class II genes that is expressed in immune cells and functions as a key regulator of immune response to foreign antigens, as well as a discriminator of self from non-self antigens [20]. From a genetic perspective, a growing number of association studies have implicated the *HLA*-DRB1 genetic alterations in a broad range of immune-mediated diseases, including HCC [21–23].

In the medical literature, a large number of studies have been conducted to identify *HLA-DRB1* genetic variants (such as DRB1*07, DRB1*12 and DRB1*15) associated with HCC risk and clinical outcomes, but many of the findings in these studies are inconsistent and inconclusive [12, 20, 24–26]. A recent meta-analysis by Lin et al reported an ethnicity-dependent association between specific *HLA-DRB1* alleles and HCC risk [18]. This finding is somewhat reasonable considering the incidence and distribution of HCC are closely linked to environmental, dietary and lifestyle factors, as well as genetic profiles [16]. The incidence of HCC is rising worldwide, with more than half of HCC cases occurring in China [27]. With huge advances in biotechnology and this rising trend in China, a better understanding of the genetic mechanisms of HCC will provide an opportunity to inform prevention strategies by devising an effective screening method for early diagnosis of HCC patients. To shed some light on this opportunity and to increase the likelihood of identifying disease-causing loci, we in the present study genotyped 8 nonsynonymous coding polymorphisms in *HLA-DRB1* gene among 521 Han Chinese, and the striking finding of this study was that rs17879599 in exon 2, where glutamine (Gln99) is substituted for histidine (His99) at residue 99, might play an independent leading role in predisposition to the development of HCC. The exon 2 of the *HLA-DRB1* gene contains antigen-binding sites and a higher level of CpG-dinucleotide content than the other exons, which leads to a high degree of point mutations and DNA repair [28]. However, a literature search has failed to reveal any supportive evidence for the implication of rs17879599 in carcinogenesis. We therefore develop a working hypothesis that if involved, the mutation of rs17879599 via altering the binding ability of HLA-DRB1 to its antigens could provide a possible molecular mechanism to explain inter-individual variation in predisposition to HCC.

Considering the fact that hepatocarcinogenesis is a multistep, multigenic process, and it is unlikely that the modulatory effect of any single variants would be dramatic in predicting cancer risk [29–31]. This opinion therefore inspires us to further interrogate the joint association of study polymorphisms in *HLA-DRB1* gene with HCC risk. Another key finding of the present study is that carriers of 7 or more unfavorable alleles of *HLA-DRB1* gene were approximately 2 times more likely to suffer from HCC relative to those having 4 or less unfavorable alleles. There was also a significantly increased trend of having HCC with the increasing number of unfavorable alleles. This finding highlights the importance of adopting a multigenic approach to identify signature of genetic alterations as predictors of cancer risk. However, a note of caution should be sounded regarding the weaker significance magnitude of our haplotype and genetic score analyses than single-locus analyses. It is possible that the leading effect of a single locus might be diluted or masked in the presence of other loci under study. Another possibility might be due to insufficient power stemming from the small sample size involved. Even so, we had more than 90% power to detect a significant association between rs17879599 and HCC risk. Nevertheless, the precise molecular mechanism for the unfavorable profiles of study polymorphisms remains a challenging task [32, 33]. In addition, the incomplete coverage of *HLA-DRB1* gene in this study possibly cannot denote the whole gene function.

Besides, other limitations should be considered when interpreting our findings. Firstly, all study participants were enrolled from a single hospital. Our findings seem to be not due to population stratification, taking into account the facts regarding the homogeneity of study population (the Han nationality) and conformity to the Hardy-Weinberg equilibrium of all study polymorphisms. Secondly, as only bi-allelic polymorphisms within *HLA-DRB1* gene were assessed while disregarding the high-resolution *HLA-DRB1* alleles, it is clearly insufficient to unravel the genetics underpinnings of this gene. Thirdly, this study is retrospective in design, and association of *HLA-DRB1* genetic variation with the incidence of intrahepatic recurrence is precluded. Fourthly, HCC patients and controls were not frequency matched on age and gender, which might introduce a selection or confounding bias. However, after adjusting for these confounders, statistical significance is undoubtedly strengthened. Fifthly, since this study was carried out in Han Chinese, we are reticent in generalizing conclusions to other ethnic or racial groups in view of the ethnicity-dependent genetic predisposition to HCC as suggested by Lin et al [18]. We fully agree that a large-scale study in Chinese or other ethnic groups must be conducted to further reinforce the findings of this preliminary study.

In summary, our findings provide support for an independent leading contribution of rs17879599 in the 2^nd^ exon of *HLA-DRB1* gene to HCC risk in Han Chinese. Our findings further suggest that the mutation of rs17879599 might be responsible for the binding ability of HLA-DRB1 to its antigens. For practical reasons, *HLA-DRB1* genetic alterations might have the potentials of being used as a biomarker and a molecular target for therapeutic intervention.

## MATERIALS AND METHODS

All study participants were consecutively enrolled from the Department of Gastroenterology at The First Hospital of Qiqihar City between June 2013 and October 2015. This study included 257 HCC patients who were newly diagnosed, histopathologically confirmed or previously untreated, and 264 cancer-free controls. All controls reported a negative history of any malignancies except for non-melanoma skin cancer. No restrictions on age, gender and cancer-stage were placed at enrollment. All participants were requested to complete a self-designed questionnaire covering demographics, lifestyle factors and medical histories, and they provided a fasting blood sample stored in vapor phase liquid nitrogen until batch genotyping. All participants were of Han Chinese descent, and they agreed to participate in this study by signing written informed consents. The study protocol complied with the Declaration of Helsinki, and it was approved by the Ethics Committee of Qiqihar Medical University.

For both HCC patients and controls, data on age, gender, cigarette smoking, alcohol drinking, diabetes mellitus and hepatitis infection were collected. In addition for HCC patients, serum concentrations of AFP, GI and CEA were measured according to standard protocols at the Clinical Laboratory of The First Hospital of Qiqihar City.

The selection of eligible study polymorphisms in *HLA-DRB1* gene was based on the following scenarios: (i) it must be exonic polymorphisms; (ii) it must be nonsynonymous and bi-allelic polymorphisms; (iii) the minor allele frequency must be greater than 5% but less than 15% according to the NCBI database of Single Nucleotide Polymorphism (dbSNP) (http://www.ncbi.nlm.nih.gov/SNP/). In total, eight polymorphisms in *HLA-DRB1* gene satisfied these scenarios simultaneously, including rs199514452 (c.31T > A, p.Cys11Ser), rs201540428 (c.34A > T, p.Met12Leu), rs201614260 (c.84G > C, p.Leu28Phe) in exon 1, rs17879702 (c.133C > T, p.His45Tyr), rs17880292 (c.256G > A, p.Val86Ile), rs17879599 (c.299G > C, p.Gln99His), rs17424145 (c.341T > C, p.Val114Ala) in exon 2 and rs35445101 (c.790T > C, p.Phe264Leu) in exon 6.

Genomic DNA was prepared from peripheral blood leukocytes using the TIANamp Blood DNA Kit (Tiangen Biotect (Beijing) Co., China). Genotypes of each study polymorphism were determined by polymerase chain reaction - ligase detection reaction (PCR-LDR) method [34]. The PCR primers and LDR probes are available upon request. The accuracy of PCR-LDR method was internally validated by re-genotyping 20 randomly selected DNA samples using this method under the blindness of the case-control status, and the repeated results were completely identical.

Continuous data are expressed as mean (standard deviation) or median (inter-quartile range: 25th percentile to 75th percentile). Categorical data are expressed as counts or percentages. Two-group comparisons were conducted by the *t*-test for continuous data and by the χ^2^-test for categorical data. Considering the small number of mutant homozygous genotype for some polymorphisms, the Fisher's exact test was used for genotype comparisons between patients and controls based on a 2 × 3 table. For allele comparisons, the χ^2^-test was employed based on a 2 × 2 table. Conformity to the Hardy-Weinberg equilibrium was tested by the χ^2^-test in controls. Each polymorphism was entered into a multivariate binary Logistic regression model by considering the potential confounding impact of age, gender, smoking, drinking and hepatitis infection. To examine the joint association of eight polymorphisms in *HLA-DRB1* gene with HCC risk, a haplotype analysis was first undertaken by the HAPLO.STATS software version 1.7.1 to estimate the frequencies of derived haplotypes and their risk prediction for HCC with and without adjusting for confounding factors as mentioned above. Then, a genetic score was created to examine the cumulative impact of eight study polymorphisms on the basis of the number of unfavorable alleles carried by each participant. With zero, one or two unfavorable alleles possible for each polymorphism, the genetic score of eight polymorphisms for each participant theoretically ranges from 0 to 16. Given the limited number of participates with some scores, genetic score was *a prior* collapsed into quartiles and compared between patients and controls. Risk prediction for HCC was expressed as OR and 95% CI. A 2-sided *P* value of less than 0.05 was considered statistically significant unless otherwise indicated. Where appropriate, *P* values were corrected for multiple comparisons using the Bonferroni correction test. All statistical analyses were completed with the IBM SPSS Statistics for Windows (version 20.0, Armonk, NY: IBM Corp.) and the R project version 2.8.1 (http://r-project.en.softonic.com/). The power to detect statistical significance was calculated by the PS: Power and Sample Size Calculation software version 3.0 (Copyright ^©^ 1997–2009 by William D. Dupont and Walton D. Plummer) [35].

The linkage disequilibrium profiles between eight study polymorphisms were investigated by the Haploview software version 4.2 (http://www.broadinstitute.org/scientific-community/science/programs/medical-and-population-genetics/haploview/downloads). The linkage magnitude between polymorphisms was qualified by the *D*' and logarithm of odds (LOD), and the linkage plot was displayed according to the standard color scheme.

## Abbreviations

HLA: human leukocyte antigen
HCC: hepatocellular carcinoma
OR: odds ratio
95% CI: 95% confidence interval
AFP: alpha-fetoprotein
GI: glucose intolerance
CEA: carcinoembryonic antigen
dbSNP: database of Single Nucleotide Polymorphism
PCR-LDR: polymerase chain reaction - ligase detection reaction
LOD: logarithm of odds.
